# LTP of inhibition at PV interneuron output synapses requires developmental BMP signaling

**DOI:** 10.1038/s41598-020-66862-5

**Published:** 2020-06-22

**Authors:** Evan Vickers, Denys Osypenko, Christopher Clark, Zeynep Okur, Peter Scheiffele, Ralf Schneggenburger

**Affiliations:** 1 Laboratory of Synaptic Mechanisms, Brain Mind Institute, School of Life Science, École Polytechnique Fédérale de Lausanne (EPFL), 1015 Lausanne, Switzerland; 2 Biozentrum, University of Basel, 4056 Basel, Switzerland; 3 Present Address: Institute of Neuroscience, University of Oregon, Eugene, OR 97403 USA; 4 Present Address: Institute for Regenerative Medicine, University of Zürich, 8952 Schlieren, Switzerland

**Keywords:** Long-term potentiation, Spike-timing-dependent plasticity, Synaptic plasticity, Synaptic transmission, Neuroscience, Synaptic development

## Abstract

Parvalbumin (PV)-expressing interneurons (PV-INs) mediate well-timed inhibition of cortical principal neurons, and plasticity of these interneurons is involved in map remodeling of primary sensory cortices during critical periods of development. To assess whether bone morphogenetic protein (BMP) signaling contributes to the developmental acquisition of the synapse- and plasticity properties of PV-INs, we investigated conditional/conventional double KO mice of BMP-receptor 1a (BMPR1a; targeted to PV-INs) and 1b (BMPR1a/1b (c)DKO mice). We report that spike-timing dependent LTP at the synapse between PV-INs and principal neurons of layer 4 in the auditory cortex was absent, concomitant with a decreased paired-pulse ratio (PPR). On the other hand, baseline synaptic transmission at this connection, and action potential (AP) firing rates of PV-INs were unchanged. To explore possible gene expression targets of BMP signaling, we measured the mRNA levels of the BDNF receptor TrkB and of P/Q-type Ca^2+^ channel α-subunits, but did not detect expression changes of the corresponding genes in PV-INs of BMPR1a/1b (c)DKO mice. Our study suggests that BMP-signaling in PV-INs during and shortly after the critical period is necessary for the expression of LTP at PV-IN output synapses, involving gene expression programs that need to be addressed in future work.

## Introduction

The neocortex of mammals contains specific classes of excitatory and inhibitory neurons^1–4^. Amongst the inhibitory interneurons, PV-INs can sustain high - frequency AP firing, and show fast membrane potential signaling and temporal precision at their input - and output synapses^5^. PV-INs form output synapses largely on soma-near compartments of principal neurons^6^, and the resulting GABA release causes well-timed inhibition of cortical principal neurons^7,8^. Furthermore, the output synapses of PV-INs can undergo long-term potentiation^9–11^ as well as long-term depression (LTD; ref. ^11^). Plasticity of inhibition, in part provided by PV-INs, has been related to critical period plasticity in the visual^9,12,13^, somatosensory^14^ and auditory cortex^11^. It is likely that the physiological properties of PV-INs are gradually acquired during postnatal development^15^, driven by specific gene expression changes^16^. Nevertheless, little is known about the molecular mechanisms which determine the developmental acquisition of the physiological properties of PV-INs, including their fast firing properties, synaptic connectivity, and the plasticity at their output synapses.

Here, we investigate a possible role of BMP-receptor signaling for the development of these functional properties of PV-INs. BMPs are members of the TGF-beta superfamily of growth factors, with widespread roles for the embryonic development and patterning of various mammalian tissues^17–19^, including the nervous system^20,21^. In the mammalian CNS, BMP-receptors (BMPRs) and their ligands are expressed up to early adulthood^22–24^, suggesting that BMP signaling fulfills further roles in later brain development. Indeed, a role for BMP signaling in the elimination of excitatory synapses^25,26^ and in the development of mono-innervation at a large excitatory connection in the auditory brainstem, has been reported^27,28^. Earlier genetic studies showed that BMP signaling drives the growth of motor nerve terminals at the *Drosophila* neuromuscular junction^29–31^. Together, these studies suggest a role for BMP signaling in guiding the establishment of specific synaptic connectivity at excitatory connections in the mammalian brain and in the periphery.

Evidence for a role of BMP signaling in the development of inhibitory interneurons is also emerging. A previous study showed that BMP-signaling in the OLIG lineage of neuronal/oligodendrocyte precursors determines the number of oligodendrocytes and Calbindin-positive interneurons^32^, and it was shown that exogenous BMP4 can act on PV-INs or their precursors to contribute to the morphological differentiation of PV-INs^33^. However, it is unknown whether BMP signaling in PV-INs is necessary for the development of the functional properties of this class of interneurons. Here, we use genetic tools, patch-clamp recordings and single-cell gene expression analyses to address this question.

## Results

### Fast firing properties of PV-INs are largely independent of BMP-signaling

Mature PV-INs can sustain high-frequency AP firing, and show fast release kinetics and spike-timing dependent plasticity at their output synapses (see Introduction). To investigate whether the developmental acquisition of these functional properties depends on BMP-signaling in PV-INs, we genetically deleted two critical BMP-type 1 receptor subunits, BMPR1a and BMPR1b. We interbred a conventional BMPR1b KO mouse^34^ with a conditional BMPR1a KO mouse (BMPR1a^lox/lox^; ref. ^35^, targeted to PV-INs by the use of PV^Cre^ mice; ref. ^36^). To facilitate analysis, PV-INs were genetically labelled with a tdT reporter line (Ai9; see Materials and Methods). In the auditory cortex, onset of Cre-mediated recombination in PV^Cre^ mice occurs at ~P13. Therefore, we focused our analysis to an age of P19–P24. This age corresponds to a developmental period shortly after the critical period for the remodeling of sound frequency representation in primary auditory cortex at P11–P14^37,38^. We assume that in (c)DKO mice, the removal of BMPR1a in the Cre-expressing PV-INs will, in the background of BMPR1b^−/−^ mice, lead to an arrest of BMP-signaling.

**Figure 1:**
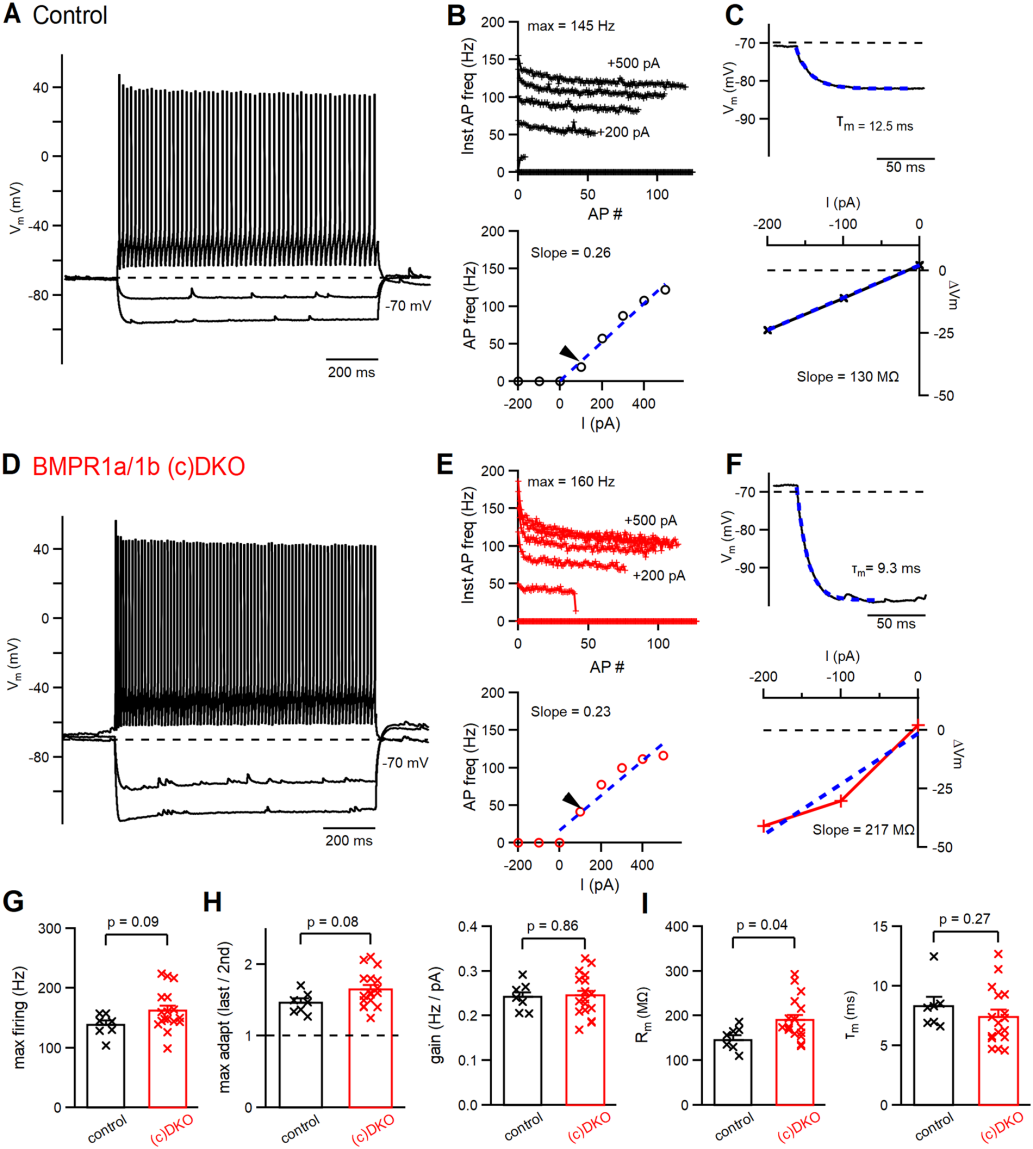
AP-firing properties and passive membrane properties of PV-INs are largely unchanged in BMPR1a/1b (c)DKO mice. **(A)** AP-firing of a PV-IN in a control mouse of age P22, in response to 1-s current steps to −200, −100 and +200 pA. (**B**) Instantaneous AP frequency (top) and plot of AP-frequency as a function of injected current (bottom). (**C**) Exponential fit (dashed blue line) to the membrane potential (V_m_) relaxation caused by a −100 pA current injection, to determine the membrane time constant (τ_m_; top), and plot of the steady-state V_m_ value as a function of the injected current to determine membrane resistance (slope = 130 MΩ in this example). The data in (**A**–**C**) are from the same recording. (**D**–**F**) Same as (**A**–**C**), but for a recording of a PV-IN in a BMPR1a/1b (c)DKO mouse of age P19. (**G**) Individual - and average data of the maximal AP firing frequency in control mice (left, black data points) and in BMPR1a/1b (c)DKO mice (right, red data points). (**H**) Individual - and average data for maximal adaptation (left), and for the firing rate gain (right). The latter was calculated as the slope in the plots of AP-firing versus injected current (**B**,**E**, bottom). (**I**) Individual - and average data for membrane resistance (left) and membrane time constant (right). For the number of recordings (n) and number of mice (N), and the statistical tests used, see Results.

We recorded tdTomato-positive PV-INs in slices of primary auditory cortex of BMPR1a/1b (c)DKO mice, and in control littermate mice. Control mice had at least one functional allele of BMPR1a and - 1b (see Materials and Methods). At the age investigated here, PV-INs in control mice exhibited high-frequency AP firing upon positive current injection, with maximal firing rates of 138 ± 7 Hz (Fig. 1A,B,G; n = 7 PV-INs). In PV-INs from BMPR1a/1b (c)DKO mice, the maximal firing frequency was higher (162 ± 8 Hz; Fig. 1D,E,G), but this trend did not reach statistical significance (p = 0.09; Fig. 1G; n = 7 and n = 17 recordings from N = 7 control - and N = 13 (c)DKO mice). Furthermore, curves of instantaneous AP-frequency versus AP number, and of time-averaged AP frequency versus injected current amplitude appeared similar between control- and (c)DKO mice (Fig. 1B,E). Accordingly, neither the maximal adaptation, nor the firing rate gain (i.e. slope of AP frequency versus injected current) were significantly different in BMPR1a/1b (c)DKO mice as compared to control mice (Fig. 1H; p = 0.08 and p = 0.86 respectively). Thus, fast AP firing of PV-INs, a property which must be acquired developmentally before the age recorded here, was not affected in BMPR1a/1b (c)DKO mice.

We furthermore analyzed the membrane resistance and membrane time constant using negative current injections. The membrane resistance was larger in BMPR1a/1b (c)DKO mice as compared to control (Fig. 1C,F), but this effect was moderate (an increase of ~25%), and on the edge of statistical significance (p = 0.04; Fig. 1I, left). The membrane time constant was not different between the two genotypes (average values of ~ 8 ms in both genotypes; Fig. 1I, right; p = 0.27, Mann-Whitney’s test). Taken together, neither the developmental acquisition of the high AP firing frequency in PV-INs, nor of their fast, subthreshold membrane potential signaling seemed to depend strongly on BMP-receptor signaling in these cells. Nevertheless, we cannot exclude that differences appear in BMPR1a/1b (c)DKO mice with further development.

### Changes in release probability at the output synapses of PV-INs

**Figure 2:**
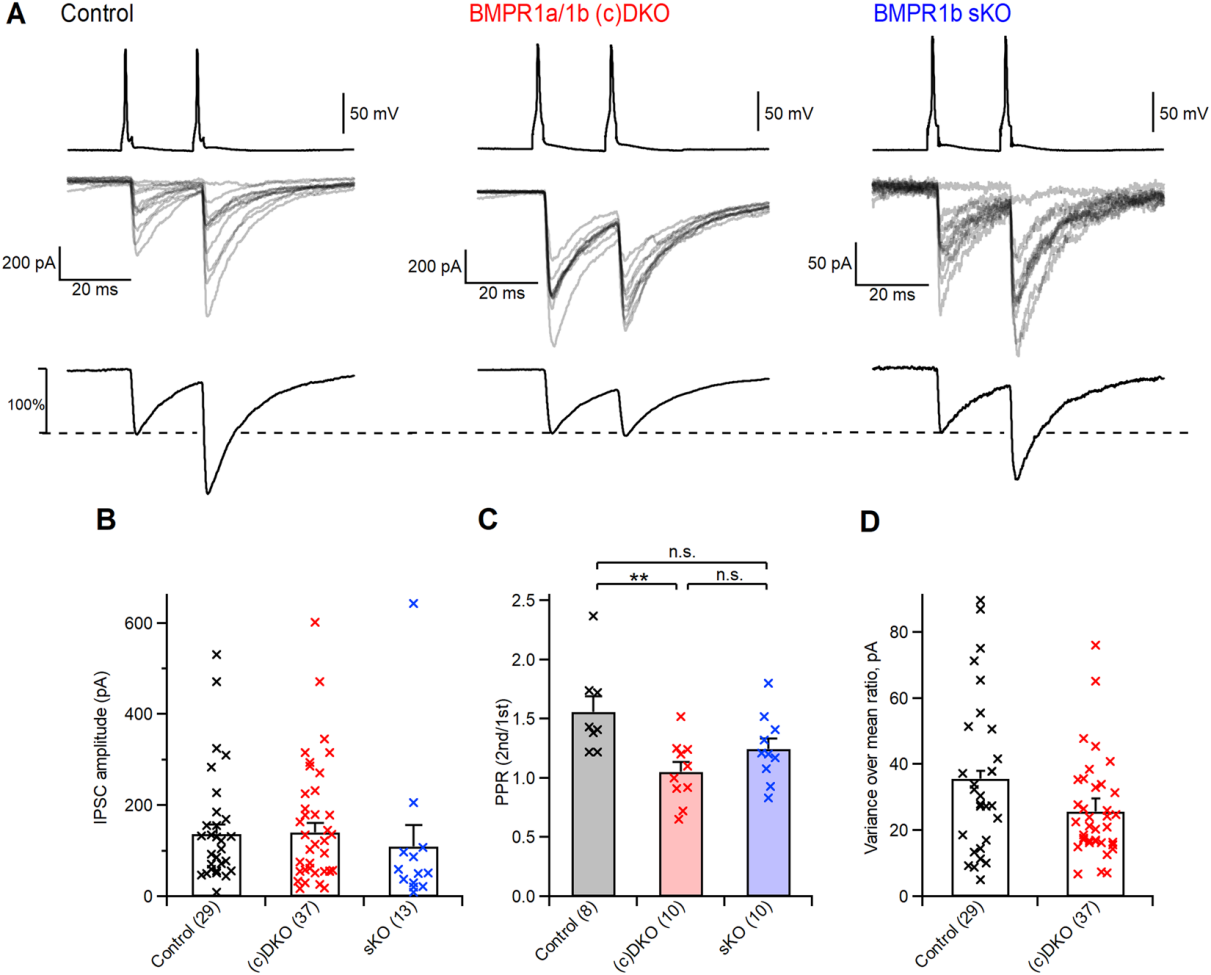
Knock-out of BMP-type1 receptors in PV-INs leads to an increased release probability, but does not affect baseline synaptic transmission at PV-IN output synapses. **(A)** APs evoked by brief current injections in PV-INs (V_m_ traces on top), and the resulting IPSCs in a postsynaptic principal neuron of layer 4 (middle). The bottom panels show peak-normalized IPSCs averaged from a larger number of stimulations (n = 40–60). From left to right, example paired recording from a control mouse at P23, from a BMPR1a/1b (c)DKO mouse at P21, and from a BMPR1b sKO at P22. (**B**) Individual - and average values of unitary IPSC amplitudes recorded in paired recordings in control mice (left); in BMPR1a/1b (c)DKO mice (middle) and in BMPR1b sKO mice (right). A Kruskal-Wallis test did not find a significant difference between the groups (see Results). (**C**) Individual - and average data for paired-pulse ratio (PPR) in a subset of recordings in which paired stimulation was applied, for the same genotypes as in (**B**). (**D**) Individual- and average values of the IPSC variance - mean ratio of the IPSC peak amplitudes, in control mice (left) and in BMPR1a/1b (c)DKO mice (right). In panels (B–D), the number of recordings (“n”) is indicated on the x-axis; for the number of animals (“N”) see Results. For statistical tests and p-values, see Results. **p < 0.01; n.s., not significant.

We next investigated whether the properties of synaptic transmission at inhibitory synapses formed by PV-INs onto L4 principal neurons in auditory cortex are altered in the (c)DKO mice. We performed paired whole-cell recordings; PV-INs were identified by their tdTomato fluorescence, and postsynaptic principal neurons by their characteristic morphology and by their AP firing properties^11^. We found that unitary IPSCs in control mice covered a large range of amplitudes across paired recordings (~10–500 pA), with an average amplitude of 149 ± 23 pA (n = 29; N = 18; Fig. 2A,B, left), in good agreement with previous work^11^. In BMPR1a and -1b (c)DKO mice, the unitary IPSC amplitude also covered a large range, with an average value of 157 ± 21 pA (n = 37; N = 24), that was indistinguishable from the IPSC amplitude in control mice. We furthermore investigated BMPR1b single KO mice (sKO). This was necessary to control for non-specific effects that might result from the constitutive deletion of BMPR1b from cells other than PV-INs in the cortical network. The unitary IPSC amplitude in BMPR1b sKO mice was 109 ± 46 pA (n = 13 and N = 6), and a Kruskal-Wallis test reported no significant effect of genotype across the three groups (p = 0.99, Fig. 2B). Thus, the baseline strength of synaptic transmission at the PV-IN to principal neuron synapse was unchanged upon removal of BMPR1a and -1b from PV-INs.

In a subset of recordings, we evoked a second presynaptic AP at an interval of 20 ms to study the paired-pulse ratio of synaptic transmission, as an indicator of presynaptic release probability (PPR; defined as IPSC2/IPSC1). We found that PPR was decreased in BMPR1a/1b (c)DKO mice (Fig. 2A, bottom; Fig. 2C). ANOVA showed that PPR was significantly different across the three genotype groups (p = 0.0073). Further post-hoc statistical testing showed that (c)DKO mice and control mice had significantly different PPR (p = 0.005; Tukey’s post-hoc test). This finding suggests that the release probability at PV-IN output synapses of (c)DKO mice is increased. In BMPR1b sKO mice, the PPR was in-between the values for control mice and (c)DKO mice, but not significantly different from neither of them (p = 0.1 and 0.35 respectively; Tukey’s post-hoc test). These data indicate that the conditional removal of BMPR1a from PV-INs (in the background of the BMPR1b sKO mice) caused the observed changes in PPR.

**Figure 3:**
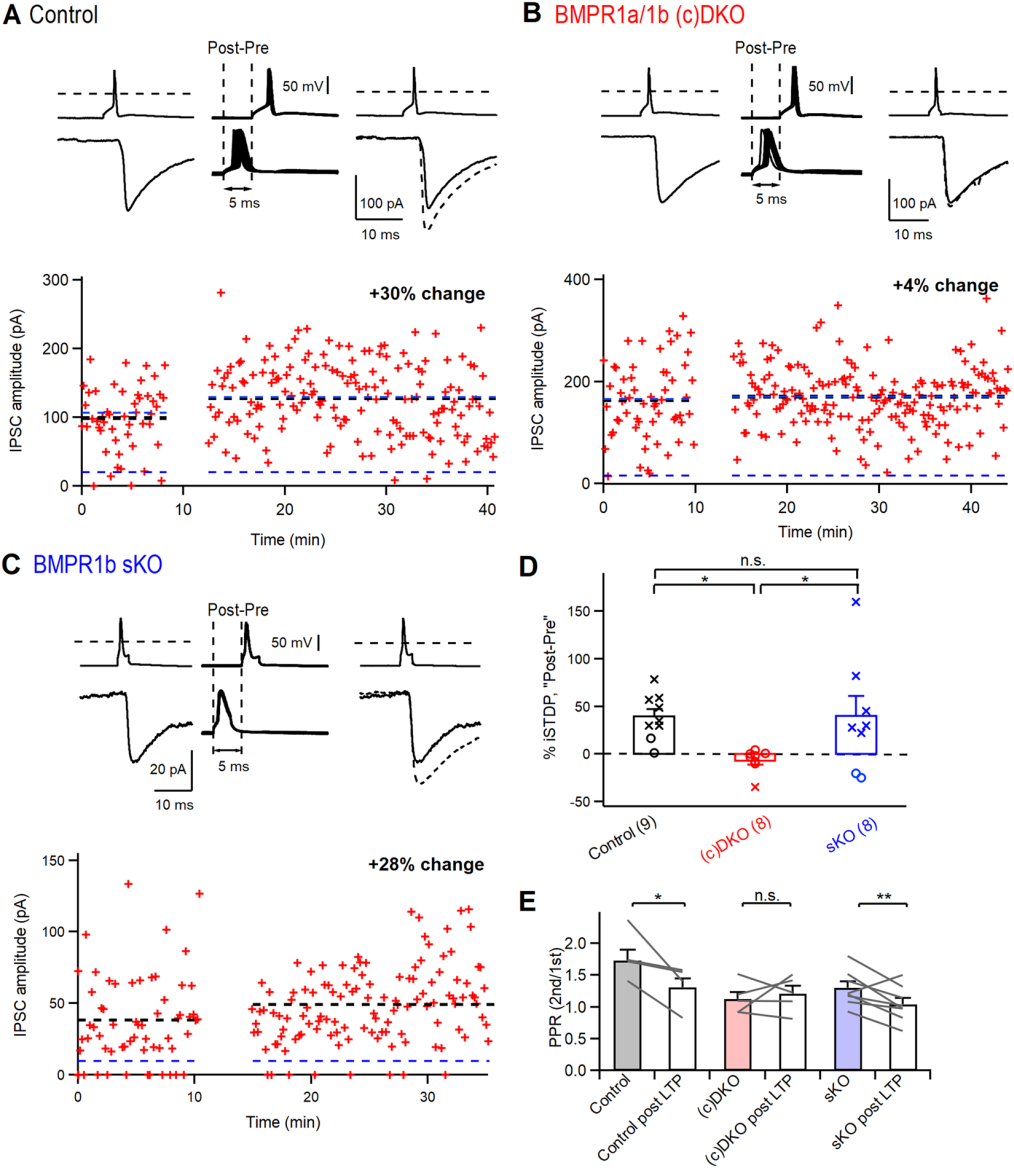
Knock-out of BMP-type 1 receptors in PV-INs impairs spike-timing dependent LTP at PV-IN output synapses. **(A**–**C)** Example of spike-timing dependent plasticity experiments with “post - pre” AP stimulation in paired recordings from a control mouse at P21 (**A**), from a BMPR1a/1b (c)DKO mouse at P20 (**B**), and from a BMPR1b sKO mouse at P21 (**C**). The traces on the top of each panel show presynaptic APs (in mV; top), and postsynaptic IPSCs during baseline (left, in [pA]), pre- and postsynaptic APs during the induction of spike-timing dependent plasticity (middle, in mV), and again IPSCs and presynaptic APs after the induction period (right, in [pA], same scale as left). Continuous IPSC traces represent average IPSCs during baseline; dashed IPSC traces represent the average IPSCs during the post-induction period. The plots on the bottom are IPSC stability plots. Thick dashed lines represent the average IPSC amplitude for baseline - and post-induction times. The amount of spike-timing dependent plasticity for each example is indicated. The lower thin dashed lines represent the threshold amplitude below which IPSCs were regarded as failures (see ref. ^11^). (**D**) Individual - and average values of spike-timing dependent plasticity measured in control mice (left, black data points), in BMPR1a/1b (c)DKO mice (middle, red data points), and in BMPR1b sKO mice (right, blue data points). Significantly - and non-significantly changed IPSC amplitudes are indicated by cross - and open symbols, respectively (t-test; p < 0.05 and p > 0.05 respectively). For the statistics of the group comparisons, and for n and N numbers, see Results. (**E**) Individual - and average values of PPR for a subset of recordings in which paired presynaptic stimuli were given. For each genotype, PPR under baseline conditions (left bar with color), and following the induction of spike-timing dependent plasticity (right open bar) is given. Left two bars, data from n = 4 recordings in control mice; middle two bars, data from n = 5 recordings in BMPR1a/1b (c)DKO mice; right two bars, data from n = 8 BMPR1b sKO mice. **p < 0.01; *p < 0.05; n.s., not significant.

An increased release probability is expected to cause an increased unitary IPSC amplitude, if other quantal parameters of transmission had been unchanged. Nevertheless, in the overall dataset, the unitary IPSC amplitude was indistinguishable between control-, and BMPR1a/1b (c)DKO mice (see above; Fig. 2B). A masking of the effects of a changed release probability could be caused by small opposing effects of either the quantal size q, and/or of the readily-releasable vesicle pool available at the PV-IN to L4 principal neuron connection. To test for such changes, we performed a variance - mean analysis, using baseline IPSC data obtained at stimulation frequency of 0.1 Hz (see below, Fig. 3A–C for examples). We found that the variance - mean value was 36 ± 4 pA (n = 29 and N = 18) and 26 ± 2 pA (n = 37 and N = 24) in control- and BMPR1a/1b (c)DKO mice, but this trend did not reach statistical significance (p = 0.1; Mann-Whitney test). In principle, a smaller variance - mean ratio would indicate a lower quantal amplitude q. Nevertheless, a small decrease in IPSC variance would also be expected for a reduced readily-releasable pool^39,40^.

### Deficient spike-timing dependent iLTP in the absence of BMP-signaling in PV-INs

We next investigated spike-timing dependent long-term plasticity at the inhibitory synapses formed by PV-INs onto L4 principal neurons^11^. After establishing a baseline unitary IPSC amplitude for a given paired recording (Fig. 3A, left), we applied repeated AP stimuli in the postsynaptic - then presynaptic order at an interval of 5 ms (50 times, 0.2 Hz; “post-pre induction”; Fig. 3A, middle). This induced a long-lasting potentiation of the unitary IPSC amplitude by 40 ± 8% (n = 9, N = 6; Fig. 3D, left), similar to previous results^11^. In the BMPR1a/1b (c)DKO mice, the same protocol failed to induce LTP of inhibition in most recordings (Fig. 3B), and occasionally caused long-term depression (LTD) of inhibition. Because the amount of plasticity observed in (c)DKO mice was close to zero in many recordings, we next performed a statitical analysis to determine if the change in IPSC amplitude after the induction protocol was significant. This revealed that in recordings from BMPR1a/1b (c)DKO mice, the change in IPSC amplitudes was in most cases not significant (p > 0.05, unpaired t-test; see Fig. 3D, dataset in the middle, open circles; n = 7 out of n = 8 recordings). Conversely, in the recordings from control mice, significant iLTP was observed in n = 8 out of n = 9 recordings (p < 0.05, unpaired t-test; Fig. 3D, data set on the left, cross symbols). The group average revealed a decrease in IPSC amplitude to −6 ± 4% in (c)DKO mice (n = 8, N = 6; Fig. 3D, middle). In the BMPR1b sKO mice, LTP was indistinguishable from that of control mice (Fig. 3C,D, right; n = 8, N = 5). ANOVA reported a significant effect of genotype on iLTP (Fig. 3D; p = 0.027). Tukey’s post-hoc test found that the magnitude of iLTP was significantly smaller in BMPR1a/1b (c)DKO mice as compared to control mice (p = 0.044), and as compared to the BMPR1b sKO mice (p = 0.046; Fig. 3D). These findings suggest that BMP-receptor signaling in PV-INs at ~ P15 - P20 is a pre-requisite for the development of cellular mechanisms that enable spike-timing dependent LTP at PV – IN output synapses.

In a subset of the recordings, we again applied paired stimuli to measure possible changes in PPR after the induction of LTP. In the control mice, the PPR was significantly reduced after the induction of LTP (p = 0.046, n = 4; Fig. 3E, left), consistent with a presynaptic locus of expression for LTP of inhibition^11^. In (c)DKO mice, the PPR was not significantly changed after the induction protocol (p = 0.39, n = 5; Fig. 3E, middle), consistent with an absence of LTP in this genotype (Fig. 3D). Furthermore, the PPR before induction was smaller than in the control mice (see also Fig. 2C). Finally, in BMPR1b sKO mice, the PPR was decreased by LTP induction (p = 0.008, n = 8 Fig. 3E, right). These findings are consistent with the notion of an increased release probability in BMPR1a/1b (c)DKO mice (Fig. 2C), and that this increased release probability could occlude the expression of LTP at the output synapses of PV-INs.

### Expression of TrkB is unchanged in PV-INs of BMPR1a/1b (c)DKO mice

**Figure 4:**
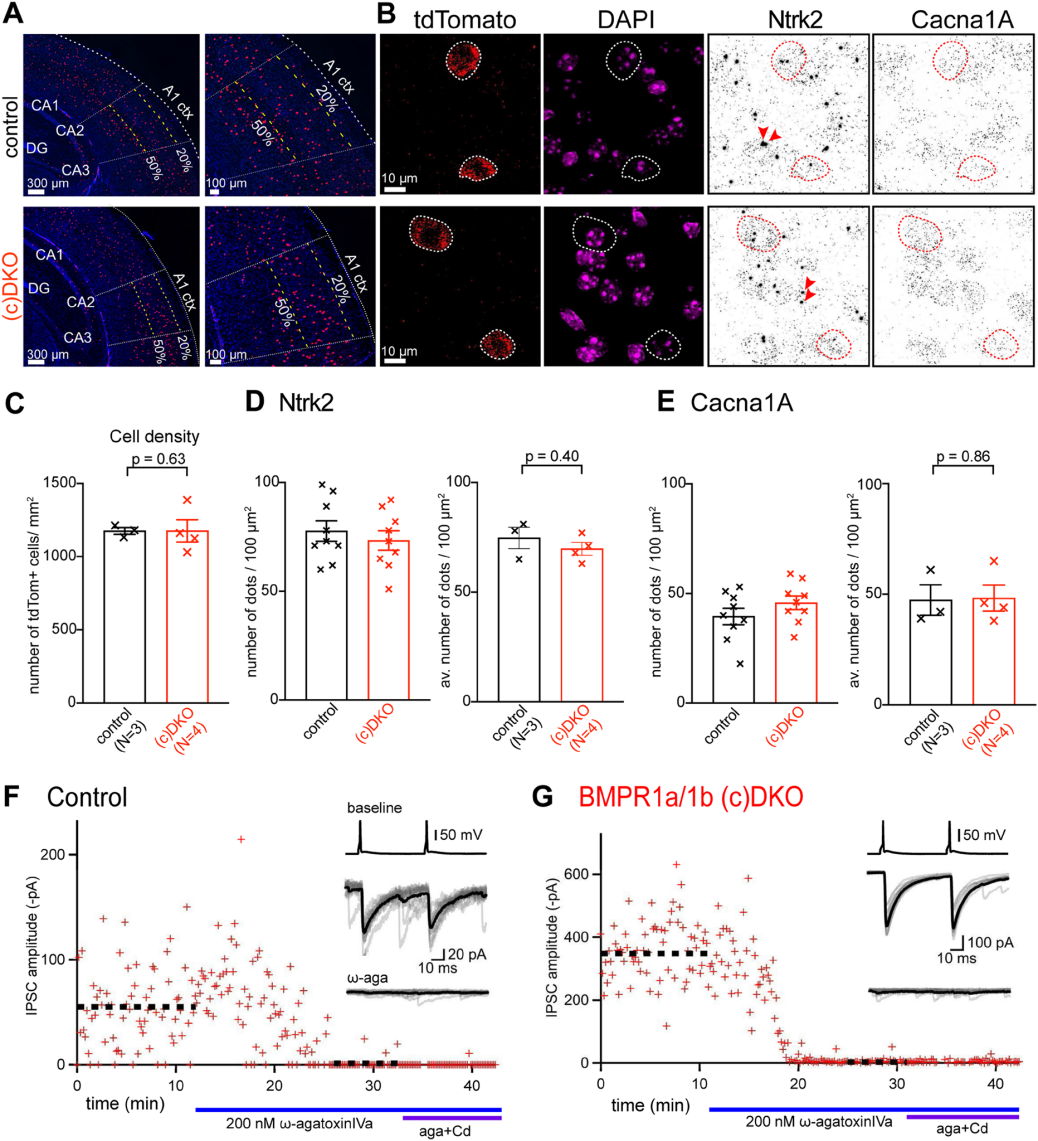
Expression levels of TrkB and P/Q-type Ca^2+^ channels are unchanged in PV-INs of BMPR1a/1b (c) DKO mice. **(A)** FISH images of coronal brain sections at two magnifications (left, and right), for a control mouse at P25 (bregma, ~−2.1 mm), and for a BMPR1a/1b (c)DKO mouse at P25 (bregma, ~−2.4 mm). The red and the blue channels show the FISH probe for tdTomato (indicating PV-INs) and DAPI, respectively. “A1 ctx”, auditory cortex. (**B**) FISH images at higher magnification for a control mouse at P25 (top) and for a BMPR1a/1b (c)DKO mouse at P25 (bottom), showing, from left to right, the tdTomato probe channel with two PV-positive neurons each, the DAPI channel, the Ntrk2 channel, and the Cacna1A probe channel. Red arrowheads show nascent transcripts of Ntrk2 in the nucleus which were excluded from the quantifications. (**C**) The density of PV-INs within 20–50% depth of auditory cortex was unchanged between N = 3 control mice (left, black data points), and N = 4 BMPR1a/1b (c)DKO mice (right, red data points). (**D**) Left, individual - and average data of the normalized number of Ntrk2 transcript dots for n = 9 PV-INs each from a control mouse (black data points), and from a BMPR1a/1b (c) DKO mouse (red data points). Right, averaged data from N = 3 control mice (left) and from N = 4 BMPR1a/1b (c)DKO mice (right). (**E**) Similar data as in (**D**), here for Cacna1A transcript levels, the gene which codes for the α-subunit of the P/Q-type Ca^2+^ channel. (**F,G**) Example paired recordings in which the P/Q-type Ca^2+^ channel blocker ω-agatoxinIVa (200 nM) was applied, followed by the application of the same toxin plus 100 µM CdCl_2_. Note that unitary IPSCs are almost completely blocked by ω-agatoxin-IVa in both genotypes. The inset shows traces of presynaptic APs (top, only for control conditions), and of postsynaptic IPSCs before - (middle) and after application of ω-agatoxinIVa (bottom). For statistical analysis and number of recordings, see Results.

Activation of BMP-receptors modifies gene expression in target cells by SMAD-dependent signaling cascades^41^. In BMPR1a/b (c)DKO mice, we observed alterations in presynaptic release probability and of LTP at PV-IN output synapses (Figs. 2 and 3). Transmitter release at PV-IN output synapses is mediated by P/Q-type Ca^2+^ channels^42,43^, and spike-timing dependent LTP of inhibition involves retrograde BDNF signaling from principal neurons onto nerve terminals of PV-INs^11^. Thus, we probed whether the mRNA levels coding for the P/Q - type Ca^2+^ channel subunit α-1A (Cacna1A) and for the BDNF receptor TrkB (Ntrk2) might be altered in BMPR1a/1b (c)DKO mice. We used fluorescent *in-situ* hybridization (FISH) on sections from control - and (c)DKO mice; PV-INs were detected with a tdTomato FISH probe based on the Cre-dependent expression of this reporter gene in PV-INs. We found that the density of PV-INs in the auditory cortex was unchanged in BMPR1a/b (c)DKO mice as compared to control mice (Fig. 4A,C). To investigate the mRNA expression levels of Cacna1a and Ntrk2, we counted the puncta within the somata of tdTomato - positive neurons (Fig. 4B). In n = 9 cells analyzed from one control - and one (c)DKO mouse each, we did not observe a difference in the number of puncta normalized by cell surface (Fig. 4D, left). Repeated measurements in n = 32 cells from N = 3 control mice, and in n = 35 cells from N = 4 BMPR1a/1b (c)DKO mice, did not indicate a significant change in the expression of Ntrk2 in PV-INs (Fig. 4D right, p = 0.4; Mann-Whitney test). Similarly, the mRNA expression of Cacna1a was unchanged between control mice, and BMPR1a/1b (c)DKO mice (Fig. 4E; p = 0.86; Mann-Whitney test).

To further test for the functional expression of presynaptic P/Q-type Ca^2+^ channels at the nerve terminals of PV-INs, we measured unitary IPSCs in paired recordings, and applied the specific P/Q-type Ca^2+^ channel toxin ω-agatoxin-iva^44^. We found that the block of synaptic transmission by ω-agatoxin was unchanged between control - and BMPR1a/1b (c)DKO mice (97 ± 1.5%, n = 2; and 97 ± 1.0%, n = 6; p = 0.99; Fig. 4F,G). Thus, consistent with unaltered Cacna1A transcript levels, P/Q-type Ca^2+^ channels continue to be the predominant Ca^2+^ channels at the output synapses of PV-INs of BMPR1a/1b (c)DKO mice.

## Discussion

Layer 4 of sensory cortices receives information from thalamus via excitatory synapses on principal neurons, and this thalamocortical drive activates a powerful feedforward inhibition mediated by PV-INs^7,8^. In the input layers of auditory cortex, the timing of AP firing is relevant^45–47^, and marked spike-timing dependent plasticity occurs both at excitatory synapses^48^, and at inhibitory synapses formed by PV-INs onto principal neurons in layer 4 of the auditory cortex^11^. In the auditory cortex of rodents, exposure to a predominant sound frequency during a critical period at P11–P14 leads to enhanced representation of that sound frequency^11,37,38^. We previously found that at an age immediately following the critical period (P15–P22), induction protocols with opposite AP sequences (pre - post *versus* post - pre) cause opposing directions of long-term plasticity at the output synapses of PV-INs^11^. Upon further development, at an age of 4–5 weeks, however, both spike-timing sequences cause long-term *potentiation* at the PV-IN output synapses^11^. Thus, the direction of plasticity at the output synapses of PV-INs is developmentally regulated, and a symmetric learning rule of potentiation of inhibition is attained in more mature animals. A symmetric learning rule with potentiation of inhibition is, in turn, expected to stabilize neuronal networks^49^. Nevertheless, the molecular mechanisms that drive the developmental changes of long-term plasticity at inhibitory synapses have been unknown.

Previous work showed that BMP signaling occurs in developing PV-INs and contributes to the morphological differentiation of PV-INs^33^, but the effects of interrupting BMP signaling on the functional properties of PV-INs had not been studied. We used conditional genetic inactivation of BMPR1a in PV-INs, in the background of BMPR1b sKO mice, and studied these mice at P19–P24, about 7–10 days after the onset of Cre-expression in PV-INs at ~ P13. In BMPR1a/1b (c)DKO mice, there were no consistent effects on the passive membrane properties of PV-INs, nor on the high rate of AP-firing that these neurons can generate (Fig. 1). On the other hand, spike-timing dependent LTP of inhibition upon post - pre induction protocols was absent, and there was a concomitant reduction of PPR indicating an increased release probability at the output synapses of PV-INs (Figs 2, 3). In BMPR1b single KO mice, no deficits of LTP of inhibition were observed (Fig. 3D). These data show that developmental BMP-signaling in PV-INs determines presynaptic properties of the output synapses of PV-INs, including release probability, and spike-timing dependent long-term potentiation.

Given a role of BMP-signaling in determining the properties of the output synapses of PV-INs, it is tempting to speculate in which neuronal compartments of PV-INs BMP-receptors are localized, and which is the source of BMP that activates these receptors. At the drosophila neuromuscular junction, evidence for a retrograde BMP signaling direction from postsynaptic - to presynaptic compartments was obtained by genetic approaches^31,50^. However, because of a lack of suitable antibodies for immunohistochemistry of BMP-receptors, we have not been able to study the localization of BMP-type 1 receptors in PV-INs. Single-cell genome-wide expression data suggests that BMPR1b is strongly expressed in astroctyes but only weakly expressed in various neuron types, whereas BMPR1a is more strongly expressed in neurons, including PV-INs (see https://www.brainrnaseq.org/ established by ref. ^51^, and http://greenberg.hms.harvard.edu/project/gene-database/ established by ref. ^52)^. This might suggest that amongst the two type-1 BMP-receptors investigated here, BMPR1a is mainly responsible for initiating BMP-signaling in PV-INs early postnatally; however, further experiments with the single conditional KO are necessary to reinforce this conclusion.

We have previously found that LTP of inhibition involves the activation of TrkB receptors, most likely localized on the presynaptic nerve terminals of PV-INs^11^. Previous work has also shown that BDNF signaling can influence critical period plasticity by acting on inhibitory neurons in the visual cortex^53^. Furthermore, earlier *in-vitro* experiments have reported that BMPs and neurotrophins, such as BDNF and NT3, can act synergistically in neurons^54^, and that BMP2 can increase the expression of the NT3 receptor TrkC in peripheral neurons^22,55,56^. For these reasons, we started to investigate whether Ntrk2, the gene coding for TrkB, shows a misregulated expression on the mRNA level in PV-INs of BMPR1a/1b (c)DKO mice. However, we did not find significant changes in transcript levels of Ntrk2 in PV-INs of BMPR1a/1b (c)DKO mice (Fig. 4). Nevertheless, it remains possible that BMP-signaling in PV-INs regulates the expression level of TrkB on the protein level, and/or that a scaffolding protein necessary for the correct subcellular localization and function of the BDNF receptor TrkB is regulated by BMP-signaling in PV-INs.

We also analyzed the expression of Cacna1a, the gene which codes for the α-subunit of voltage-gated P/Q type Ca^2+^ channels. P/Q-type Ca^2+^ channels are expressed in PV-INs both on the soma-dendritic compartment and at the nerve terminal^42,43,57,58^. We found that the mRNA levels of Cacna1A were not changed significantly in PV-INs, and correspondingly, synaptic transmission at the output synapses of PV-INs continued to be highly sensitive to the P/Q-type Ca^2+^ channel blocker ω-agatoxin-IVa (Fig. 4). Thus, the target genes downstream of BMP- and SMAD-signaling in PV-INs need to be systematically investigated in future studies, possibly using genome-wide screening approaches. Identifying target genes whose expression is regulated by BMP-signaling in PV-INs might allow further insights into the signaling pathways that enable a presynaptic, BDNF-dependent form of LTP at the output synapses of these interneurons^11^.

PV-INs are characterized by the high AP firing frequency they can sustain^5^, a property which is acquired during postnatal development^15,16^. We did not find clear effects on the high AP firing frequency that these neurons can support, nor on the membrane time constant of PV-INs in BMPR1a/1b (c)DKO mice (Fig. 1). The input resistance of PV-INs was moderately increased in BMPR1a/b (c)DKO mice (Fig. 1I), which might suggest a slower developmental maturation towards a low membrane resistance in adult animals^15^.

Taken together, we find that developmental BMP-signaling in PV-INs determines presynaptic properties of the output synapses of these interneurons, including release probability and spike-timing dependent LTP of inhibition. A symmetric “learning rule” of long-term *potentiation* of inhibition regardless of the exact sequence of pre- and postsynaptic APs is established at ~P28 (ref. ^11^), and a symmetric learning rule is likely beneficial for the stability of neuronal networks^49^. Thus, it is possible that developmental BMP-signaling in PV-INs, which supports LTP of inhibition, is a prerequisite for certain types of homeostatic plasticity in cortical networks^9,59^. This hypothesis could be tested in future systems-level investigations of cortical function using BMP-receptor mutants.

## Materials and Methods

### Ethics statement

All experiments with laboratory mice followed the guidelines and regulations of the Swiss Federal law on the protection of animals (“Loi fédérale sur la protection des animaux”). The specific experimental procedures with laboratory mice were approved by the SCAV (Service of Consumption and Veterinary Affairs, Canton of Vaud, Switzerland; authorizations VD2063.3, VD2063.4).

Mouse pups were kept in the homecage with their mother; weaning was done at P25. One mouse at a time, at age P19 to P24, was carefully removed from the cage, and killed by decapitation either without prior anesthesia, or after a brief isoflurane anesthesia in later experiments (protocols approved by the SCAV).

### Mouse lines

We wished to inactivate BMP-signaling in PV-INs, by genetically deleting two essential BMP-type 1 receptors, BMPR1a and -1b. For this purpose, we generated a conditional/conventional double KO (DKO) mouse model of BMP-receptor (BMPR) 1a and 1b, based on an interbreeding of four mouse lines. (1) A conditional knock-out (KO) mouse line of the Alk3 gene which codes for BMPR1a (BMPR1a^lox/lox^; ref. ^35^). (2) A conventional KO mouse of the Alk6 gene which codes for BMPR1b (BMPR1b^−/−^; ref. ^34^). (3) A PV^Cre^ mouse line to drive Cre-expression in PV-INs (PV^Cre/+^; ref. ^36^). (4) A reporter mouse line driving the expression of tdTomato in Cre-expressing cells (Ai9; ref. ^60^; called here “tdT”), which was used to target recordings to PV-INs and to guide cellular gene expression analyses. In breeding pairs that gave rise to mice used here, both males and females were heterozygous for the BMPR1a locus (BMPR1a^+/lox^) and for the BMPR1b locus (BMPR1b^+/−^), and homozygous in the PV locus (PV^mut/mut^) and for the tdT transgene (tdT^mut/mut^). The offspring of these breedings therefore showed the following genotypes: (1) Homozygous (c)DKO mice with genotype of BMPR1a^lox/lox^, BMPR1b^−/−^; this genotype is expected to occur at a fraction of 1/16. (2) Homozygous wild-type mice with genotype of 1a^+/+^ [for BMPR1a^+/+^], 1b^+/+^ [for BMPR1b^+/+^]; expected at a rate of 1/16). (3) Heterozygous animals with at least one functional allele of each BMP-type 1 receptor (i.e. genotypes 1a^+/+^, 1b^+/−^ at 1/8; 1a^+/lox^, 1b^+/−^ at 1/4; 1a^lox/+^, 1b^+/+^ at 1/8). Because homozygous wild-type mice were difficult to obtain in this breeding scheme, we used mice with at least one functional allele of each BMP type 1 receptor as “wild-type” controls (all genotypes listed in group (3) above). (4) Single KO (sKO) mice for the BMPR1b (genotypes, 1a^+/+^, 1b^−/−^ at 1/16; 1a^+/lox^, 1b^−/−^ at 1/8); these mice were used to control for compensatory networks effects that might result from the constitutive inactivation of the BMPR1b allele (see Results). (5) Single conditional KO mice of the BMPR1a allele (genotypes, 1a^lox/lox^, 1b^+/−^ at 1/8; 1a^lox/lox^, 1b^+/+^ at 1/16) - these mice were not used for experiments. We observed that the genotype fractions roughly conformed with the expected Mendelian ratios, although in some cases fewer (c)DKO mice than expected seemed to be present by ~P6. For this reason, in early breedings, at least one parent was homozygous for BMPR1a^lox^. Although (c)DKO mice could only be obtained at low numbers, the above breeding scheme had the advantage that all three mouse genotype groups were obtained from the same breedings (“wild-type” control mice, (c)DKO mice, and BMPR1b sKO mice).

### Slice preparation and patch-clamp electrophysiology

We made patch-clamp recordings of PV-INs identified by their tdTomato fluorescence, either alone or together with a recording of a connected principal neuron, using an EPC10/2 patch-clamp amplifier (HEKA elektronik, Germany, see https://www.heka.com/). Recordings were made in slices of auditory cortex of young mice (P19–P24), at a depth of 30–50% from the cortical surface, which we regard as layer 4 (ref. ^11^). By targeting tdTomato-positive neurons using PV^Cre^ mice, we assume that we target fast-firing, PV-positive basket cells, which are an abundant interneuron population in layer 4 of mouse auditory cortex^61^. However, because chandelier cells also express PV (ref. ^62^), and have similar AP firing properties as basket cells^63^, it remains possible that a minority of the sampled cells represent chandelier cells. We note, however, that in primary auditory cortex and other sensory cortices of mice, only few of the more complex nerve endings of chandelier cells were detected^64^, and that in many cortical areas, the density of chandelier cells is low in layer 4.

Parahorizontal thalamocortical auditory slices (300 µm) of mouse brain were made with a vibratome (Leica VT 1200). Whole-cell patch-clamp recordings were performed at room temperature (21–23 °C), in set-ups with an upright microscope (either Olympus BX51WI, or ZEISS Axioskop 2), equipped with 60×, 0.9 numerical aperture objectives (Olympus). The tdTomato fluorescence of genetically labeled PV-INs (tdT reporter mice; see above) was excited with a Polychrome V (or Polychrome IV; TILL Photonics) monochromator at 550 nm (using a 570–613 nm emission filter). Images were detected with a CCD camera (either Retiga 2000RV or PCO Sensicam).

The patch-pipette (intracellular) solution for recording PV-INs contained (in mM): 135 K-gluconate, 10 KCl, 0.5 HEPES, 5 Na_2_-Phosphocreatine, 4 Mg-ATP, 0.3 Na_2_-GTP, 0.5 EGTA. The pH was set to 7.2 by adding KOH, the osmolarity was ~305 mOsm. The patch pipette solution for recording postsynaptic principal neurons was similar to the above solution, but contained 160 mM KCl and no K-gluconate. Because of the high intracellular [Cl^−^], IPSCs were recorded as inward currents at a holding potential of −70 mV. The extracellular solution was a standard bicarbonate-buffered solution containing 2 mM CaCl_2_ and 1 mM MgCl_2_. For the measurement of spike-timing- dependent plasticity of IPSCs, the series resistance (R_S_) of the postsynaptic recording was minimized, and R_S_ was verified regularly throughout the recording. A change in the R_S_ by more than ±50%, and above a value of 20 MOhm led to the exclusion of a recording from the final dataset.

For measurements of the AP-firing behavior and passive membrane properties in recordings of PV-INs alone (Fig. 1), 1 s current steps between −200 and +500 pA (increments of +100 pA) were applied in current-clamp experiments in single recordings. During paired recordings, unitary IPSCs between the PV-IN and a L4 principal neuron were first measured under baseline conditions, by applying short (3 ms) current steps in the PV-INs under current clamp to evoke single APs repeated every 10 s; the resulting postsynaptic IPSC was measured with a second patch-clamp amplifier under voltage-clamp at a holding potential of −70 mV. Following this baseline period, a spike-timing dependent plasticity protocol was applied, in which a postsynaptic AP was followed by a presynaptic AP (post - pre induction; see ref. ^11^). Specifically, post- and presynaptic current injections (1 nA of 3 ms duration in both recordings) were applied with a time offset of 5 ms, 50 times every 5 s.

### Fluorescent *in situ* hybridization

Fluorescence *in situ* hybridization (FISH) was performed using the RNAscope Fluorescent Multiplex Kit (ACD) protocol. Briefly, brains were dissected from P25 male and female mice and snap frozen in liquid nitrogen. Coronal sections (18 μm thick) were cut with a cryostat between Bregma −2 and −3.6 mm to include auditory cortex, adhered to Superfrost ultra plus slides (Thermo Scientific) and stored at −80 °C. Sections were fixed for 30 min in 4% PFA and treated with Protease IV. Hybridization was for 2 hours at 40 °C. The following probes (50x dilution), coupled to specific fluorophores, were used in the same hybridization reaction: *Ntrk2* (C1 # 423611; coupled to Atto 550), *Cacna1A* (C2 # 493141; Alexa 488) and *tdTomato* (C3, # 317041; Atto 647). DAPI was used to identify the nuclei. Images were acquired with an upright LSM700 confocal microscope (Zeiss) using a 40x Apochromat objective in z-stacks (19–22 images, 0.4 µm intervals), using laser lines of 405 nm (for exciting DAPI), of 488 nm (for Alexa 488), of 555 nm (for Atto 550), and of 639 nm (for Atto 647). PV-INs were identified based on the presence of the tdTomato probe signal, and the expression of TrkB and Cacna1a was analyzed in PV-INs contained in a depth of 20–50% of auditory cortex. A region of interest (ROI) was drawn to define the area of the cell soma. Thresholding for detection of the signal throughout the stacks and the number of pixels was done with Fiji software (https://fiji.sc/), using the TrackMate plugin (https://imagej.net/TrackMate). The number of puncta for each probe set were then counted with the TrackMate plugin in the ROI. The number of puncta in the ROI, normalized to the area, were used as a proxy for expression strength of a given probe. The density of tdTomato positive cells spanning from 20% to 50% depth of auditory cortex was quantified from images acquired on a Widefield Axio Scan Z1 slide scanner. Images were assembled using Fiji and Adobe Illustrator CS6 (https://www.adobe.com/uk/products/illustrator.html).

### Analysis and statistics

Analysis of electrophysiological data was made with Igor.Pro (versions 6.37 and 7.0.8.1; https://www.wavemetrics.com), using custom-written functions. The membrane time-constant (τ_m_) was measured by fitting a single exponential function to the relaxation of the membrane potential (V_m_) trace in response to a 1 s, −100 pA current step. The instantaneous AP frequency was measured from the inter-AP intervals for all pairs of subsequent APs in response to 1 s current injections. The maximal adaption was calculated as the last interspike interval (ISI) in the train divided by the second ISI in the train.

Statistical tests for the analysis of electrophysiological data were performed in GraphPad Prism 5.01 (https://www.graphpad.com/scientific-software/prism/). For repeated experiments, the number of recorded cells or cell pairs is reported as “n”, and the number of investigated mice as “N”. For experiments in which the control group and the BMPR1a/1b (c)DKO group were compared, we first performed a Shapiro-Wilk normality test to determine whether the data was normally distributed. For datasets which passed the normality test we performed an unpaired Student’s test to determine the statistical significance of the difference between the two groups. If one of the groups failed the normality test, we performed a non-parametric Mann-Whitney test for unpaired comparisons instead, as indicated in the Results. For the comparison of paired-pulse ratios (PPR) before- and after LTP induction in a given recording, we used paired Student’s t-test.

For experiments in which three groups of mice were compared, we used one-way ANOVA if all datasets passed the Shapiro-Wilk normality test. If one or more of the datasets failed the normality test, a non-parametric Kruskal-Wallis test was used, as indicated in the Results. In both cases, if the p value of the ANOVA/ Kruskal-Wallis test was below 0.05, we performed post hoc tests, corrected for multiple comparisons. For parametric one-way ANOVA, we used the Tukey’s multiple comparisons test. In case of the non-parametric Kruskal-Wallis test, we used Dunn’s multiple comparisons test. In either case, alpha was set to 0.05.

Statistical tests for the quantifications of PV-IN cell density, TrkB and Cacna1A mRNA levels were performed in GraphPad Prism 8 (https://www.graphpad.com/scientific-software/prism/). The number of dots per cell were averaged to yield an average expression level in a given control- and (c) DKO mouse for both the TrkB- and Cacna1a probe. This analysis was repeated in a total of N = 3 control mice and N = 4 (c)DKO mice, and the statistical difference was tested with a non-parametric Mann-Whitney test for unpaired comparisons.

## Data Availability

The raw data leading to the conclusions of this paper can be found at Zenodo (10.5281/zenodo.3827171).
